# Beyond the Microscope: A Technological Overture for Cervical Cancer Detection

**DOI:** 10.3390/diagnostics13193079

**Published:** 2023-09-28

**Authors:** Yong-Moon Lee, Boreom Lee, Nam-Hoon Cho, Jae Hyun Park

**Affiliations:** 1 Department of Pathology, College of Medicine, Dankook University, Cheonan 31116, Republic of Korea; vilimoon@hanmail.net; 2 Department of Biomedical Science and Engineering (BMSE), Institute of Integrated Technology (IIT), Gwangju Institute of Science and Technology (GIST), Gwangju 61005, Republic of Korea; leebr@gist.ac.kr; 3 Department of Pathology, Severance Hospital, College of Medicine, Yonsei University, Seoul 03722, Republic of Korea; CHO1988@yuhs.ac; 4 Department of Surgery, Wonju Severance Christian Hospital, Wonju College of Medicine, Yonsei University, Wonju 26492, Republic of Korea

**Keywords:** AI-assisted diagnostics, PAP smear classification, cervical cancer screening, digital pathology, healthcare insurance

## Abstract

Cervical cancer is a common and preventable disease that poses a significant threat to women’s health and well-being. It is the fourth most prevalent cancer among women worldwide, with approximately 604,000 new cases and 342,000 deaths in 2020, according to the World Health Organization. Early detection and diagnosis of cervical cancer are crucial for reducing mortality and morbidity rates. The Papanicolaou smear test is a widely used screening method that involves the examination of cervical cells under a microscope to identify any abnormalities. However, this method is time-consuming, labor-intensive, subjective, and prone to human errors. Artificial intelligence techniques have emerged as a promising alternative to improve the accuracy and efficiency of Papanicolaou smear diagnosis. Artificial intelligence techniques can automatically analyze Papanicolaou smear images and classify them into normal or abnormal categories, as well as detect the severity and type of lesions. This paper provides a comprehensive review of the recent advances in artificial intelligence diagnostics of the Papanicolaou smear, focusing on the methods, datasets, performance metrics, and challenges. The paper also discusses the potential applications and future directions of artificial intelligence diagnostics of the Papanicolaou smear.

## 1. Introduction

Cervical cancer is the fourth most common cancer among women worldwide and is mainly caused by human papillomavirus (HPV) infection, with an estimated 604,000 new cases and 342,000 deaths in 2020 [1]. HPV is transmitted through sexual contact and can be prevented by vaccination and screening. Over 90% of new cases and deaths occur in low- and middle-income countries. Women infected with human immunodeficiency virus (HIV) are six times more likely to develop cervical cancer than women without HIV infection [2]. Cervical cancer can be cured if diagnosed early and treated promptly. Therefore, the World Health Organization (WHO) recommends HPV testing as the primary screening method, followed by treatment of pre-cancerous lesions or referral for further evaluation and management of invasive cancer, and WHO has adopted a global strategy to accelerate the elimination of cervical cancer, which involves reaching the 90-70-90 targets by 2030 [3]. These targets are 90% of girls vaccinated against HPV, 70% of women screened with a high-performance test, and 90% of women with cervical disease treated [3].

**Figure 1:**
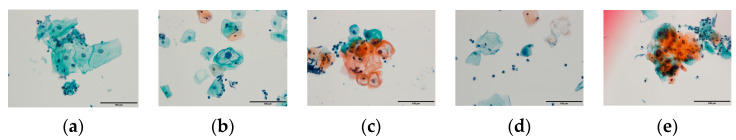
The PAP smear cytology of uterine cervical cells classified by disease progression: (**a**) normal; (**b**) atypical squamous cell of undetermined significance (ASCUS); (**c**) low-grade squamous intraepithelial lesion (LSIL); (**d**) atypical squamous cell cannot exclude HSIL (ASC-H); and (**e**) high-grade squamous intraepithelial lesion (HSIL) (segmented and labeled by the authors).

The Papanicolaou (PAP) smear is the most widely used screening method for cervical cancer. It involves collecting cells from the cervix and examining them under a microscope to identify any abnormalities (Figure 1). The PAP smear test can detect not only cervical cancer but also precancerous lesions that can be treated before they develop into cancer. The PAP smear test has been proven to be effective in reducing the mortality rate of cervical cancer by 70% [4]. However, the PAP smear test also has some limitations, such as:

- It is time-consuming and labor-intensive, as it requires trained cytotechnologists or pathologists to manually review a large number of slides.
- It is subjective and inconsistent, as different experts may have different interpretations and opinions on the same slide.
- It is prone to human errors, such as misclassification, false negatives, false positives, or missed lesions.
- It has low sensitivity and specificity, as it may fail to detect some subtle or rare abnormalities or may confuse some benign conditions with malignant ones.

To overcome these limitations, AI techniques have been applied to PAP smear diagnosis in recent years. AI techniques can automatically analyze PAP smear images and classify them into normal or abnormal categories, as well as detect the severity and type of lesions. AI techniques can also provide quantitative and objective results that are consistent and reproducible. The incorporation of AI in cervical cell screening presents numerous potential advantages, such as heightened diagnostic precision, improved efficiency, and increased patient comfort. AI algorithms can process and analyze large datasets, identifying patterns and inconsistencies that might elude human detection. Furthermore, AI-facilitated screening could lessen the necessity for invasive procedures, reducing patient discomfort and enhancing outcomes.

It is paramount to seek innovative solutions that can augment the reliability and efficiency of cervical cancer screening, thereby improving patient outcomes and lessening healthcare burdens. The impetus for this review arises from the compelling need to refine cervical cancer screening methodologies, addressing the current techniques’ drawbacks and exploring AI’s potential to transform early detection. This paper aims to provide a comprehensive review of the recent advances in AI diagnostics of the PAP smear, focusing on the methods, datasets, performance metrics, and challenges. The paper also discusses the potential applications and future directions of AI diagnostics of the PAP smear.

## 2. Performance Metrics, Datasets from Image Patches to Whole Slide Images (WSIs), from Machine Learning (ML) to Vision Transformer

In this particular section, we will carry out an extensive comparative analysis of the performance metrics and datasets that are employed in AI diagnostics of the PAP smear in the literature reviewed (Table 1). Our objective is to offer a brief summary of the essential aspects. Detecting precancerous lesions for cervical cancer in a timely manner is crucial for effective treatment and prevention. Thus, it is imperative to investigate and scrutinize the diverse techniques and methods used for PAP smear diagnostics to enhance the precision and effectiveness of detection.

In 1992, the PAPNET, the first commercial automatic screening system, was approved. However, it was only authorized as a method of re-screening for slides that were initially deemed negative by cytologists [5]. The ThinPrep^®^ imaging system (Version 1.0, Hologic, Marlborough, UK), which was approved as a commercial screening product in 2004, employs a proprietary algorithm to choose the 22 most concerning fields of view (FOV). This has reduced the workload of pathologists while also increasing the accuracy of the process [6].

The ThinPrep^®^ imaging system uses liquid-based cytology, which has several advantages over traditional methods. For instance, it is viable to obtain a more representative exemplar of the cervix, which curtails the number of mistaken negatives. The system also eliminates the need for manual fixation and staining, which can introduce variability and artifacts.

Recent studies have shown that the ThinPrep^®^ imaging system is more sensitive than traditional cytology screening. Moreover, it is additionally more duplicable, which boosts its correctness. The software is proficient in identifying subtle modifications in cells that could be a sign of cancer, enhancing its effectiveness.

Although the ThinPrep^®^ imaging system has advantages, it also has limitations. Its implementation can be expensive and specialized training is required for operators. Additionally, certain variables, such as the presence of hemoglobin or mucus, can interfere with the investigation. In the year 2008, the emergence of the FocalPoint GS imaging system marked a significant milestone in the field of cervical cytology. The system was designed to identify 10 FOV of cervical cells most likely to be abnormal, which allowed for the stratification of risk and improvement of efficiency [7]. Although there are advantages to this automated system, certain assessments indicate that its cost-effectiveness is confined and may not be suitable for use in developing countries with low to medium development [8]. Additionally, the technology has weaknesses and depends on the final manual screening process [9]. Thus, analysts continue to examine the implementation of AI technology in cervical cytology, with a focus on improving efficiency. The integration of AI technology would enable the automation of the screening process, thereby alleviating the burden on cytopathologists and elevating the accuracy and efficiency of the outcomes. Furthermore, the adoption of AI would bring about heightened cost-effectiveness throughout the entire procedure, which would be particularly beneficial for countries with limited financial resources, where affordability remains a significant concern. By embracing AI technology, the screening process would undergo a remarkable transformation, characterized by swiftness, precision, and cost-efficiency, thereby widening its accessibility to a larger population.

Chankong et al. utilized fuzzy c-means clustering technology to segment single-cell images into the nucleus, cytoplasm, and background, thereby achieving whole-cell segmentation [8].

An investigation explored a segmentation model that utilizes images extracted from a PAP smear slide. The model utilized nucleus localization to differentiate normal and abnormal cells, combined with single-cell classification algorithms. The segmentation model achieved a high level of accuracy and sensitivity, respectively, with 91.7% consisting of Mask-RCNN [10].

Recent years have witnessed a transformation in the methods employed for classification, with the majority of approaches no longer depending solely on texture feature extraction or segmentation. One such novel approach involves segmenting cervical single-cell images into the nucleus, cytoplasm, and background, and then extracting morphological features to enable automatic multi-label classification. The consequences of this strategy have been exceedingly promising, with a precision rate above 93%, suggesting the potential efficacy of this methodology in automated sorting [11].

Another innovative approach involves the extraction of seven groups of texture features of cervical cells for classification, with the support vector machine (SVM) classifier demonstrating the highest accuracy and best performance. This strategy is extremely efficient at classifying the pictures with a high level of precision. However, it is worth noting that the precision percentage of the incorporated categorizer was solely 50% at the stain plane and 60% at the unit plane, which indicates the need for further refinement and optimization [12].

Researchers are finding automated categorization methods that do not rely on an accurate segmentation algorithm to be more and more attractive. One of these methods utilizes deep learning (DL) and transfer learning to classify cervical cells. The likelihood of achieving such exceptional performance through manual extraction of deep-level features from cell images for classification, with an accuracy of 98.3%, an AUC of 0.99, and a specificity of 98.3%, is low. This highlights the significant potential of utilizing advanced machine learning techniques, such as deep learning, for improving the accuracy and efficiency of cell image analysis in various medical applications [13].

The complexity and specificity of the task at hand, as described earlier, necessitated the use of a graph convolution network for the precise classification of cervical cells. This advanced machine learning technique achieved impressive results, with precision, sensitivity, specificity, and F-measure rates of 98.37%, 99.80%, 99.60%, and 99.80%, respectively. These findings demonstrate the potential of utilizing graph convolution networks for accurate and efficient analysis of complex medical images [14].

The examination directed by Bao et al. comprises a possible companion examination of a broad populace of females, involving 700,000 people who were going through screening for cervical carcinoma. The AI-assisted cytological diagnostic system employed in the study was validated, resulting in a total coincidence rate of 94.7%. Moreover, this synchronized with a marked upsurge in sensitivity of 5.8% (3.0% to 8.6%) in comparison to manual check. This study demonstrates that the integration of AI-assisted cytological examination can significantly improve the detection and classification of cervical cells and should be considered as a potential tool for guiding triage in cervical cancer screening programs [15].

Zhu and colleagues developed an AIATBS diagnostic system that utilized ThinPrep^®^ and artificial intelligence and showed higher sensitivity than the diagnosis conducted by experienced cytologists. In fact, the AIATBS system had a remarkable sensitivity of 94.74% when detecting CIN. These discoveries have noteworthy ramifications for the domain of cervical ailment diagnosis, as they propose that AI has the potential to significantly enhance the accuracy and sensitivity of existing diagnostic techniques [16].

Chen and his colleagues carried out research on CytoBrain, a screening system for cervical cancer that employs artificial intelligence. This system utilizes deep learning technology and comprises cervical cell segmentation, classification, and human-aided diagnosis visualization modules. The study mainly focuses on cell segmentation and classification components and proposes a compact VGG network called CompactVGG as the classifier. The researchers introduced a large dataset of 198,952 cervical cell images from 2312 participants, which were categorized into positive (abnormal), negative (normal), and junk categories. The CompactVGG structure features 10 convolutional layers, 4 max pooling layers, and 2 fully connected layers, totaling 1,128,387 parameters. The independent test group found evidence of CompactVGG’s accuracy being 88.30%, sensitivity being 92.83%, specificity being 91.03%, precision being 82.26%, and F1-score being 87.04%. These outcomes surpassed the Inception v3, ResNet50, and DenseNet121 models on all metrics. Furthermore, CompactVGG demonstrated superior time and classification performance compared with existing VGG networks on the Herlev and SIPaKMeD public datasets. In conclusion, the CytoBrain system with its CompactVGG classifier has the potential to improve cervical cancer screening through its accurate and efficient performance [17].

Wei and colleagues introduced an innovative module called InCNet, which enhances the multi-scale connectivity of the network while maintaining efficiency. This module is seamlessly integrated into a lightweight model named YOLCO (You Only Look Cytopathology Once) and is specifically designed to extract features from individual cells or clusters. To evaluate their approach, the authors curated a novel dataset comprising 2019 whole slide images (WSIs) obtained from four different scanners. The dataset includes annotations for both normal and abnormal cells and is publicly accessible for research purposes.

In order to assess the performance of their method, the authors compare it with a conventional model that employs a ResNet classifier. The evaluation was conducted on 500 test WSIs. The results demonstrate that the proposed method outperforms the conventional model across most metrics, except for specificity, where the conventional model exhibits a slight advantage. The AUC score achieved by the new method is 0.872, while the conventional model obtains a score of 0.845. Moreover, the accuracy of the proposed method reaches 0.836, surpassing the accuracy of 0.824 achieved by the conventional model.

Furthermore, the authors showcase the clinical relevance of their method by illustrating its ability to detect sparse and minute lesion cells in cervical slides. This capability is particularly challenging for human experts and conventional models. The authors assert that their method has the potential to enhance the diagnosis and screening of cervical cancer, thereby contributing to improved healthcare outcomes [18].

Cheng and colleagues developed an innovative system for cervical cancer screening that utilizes deep learning techniques on WSIs. This computer-aided approach has the potential to significantly improve the accuracy and efficiency of cervical cancer screening, offering a promising new avenue for early detection and treatment. The system consists of three models, namely, low-resolution lesion localization, high-resolution cell identification/ranking, and recurrent neural network WSI classification models. To evaluate the efficacy of their system, they used 3545 WSIs from five hospitals and scanners, with 79,911 annotations. In independent testing conducted on 1170 WSIs, the system demonstrated 93.5% specificity and 95.1% sensitivity, similar to three experienced cytopathologists. The system also identified the top 10 lesion cells with a true positive rate of 88.5% on 447 positive slides, surpassing the Hologic ThinPrep^®^ Imaging System. The computational efficiency of the system is remarkable, given its ability to process giga-pixel WSIs in around 1.5 min per graphic processing unit (GPU), which illustrates its effectiveness in actual screening scenarios [19].

Wang and colleagues proposed an innovative approach for detecting cervical high-grade squamous intraepithelial lesions and squamous cell carcinoma screening in PAP smear WSIs using cascaded fully convolutional networks. This ingenious method involves a step-by-step process of utilizing fully convolutional networks to accurately identify and classify abnormalities in WSIs, which could have significant implications for improving cervical cancer screening and diagnosis. Their investigation, released in a top-tier medical journal, evaluates their proposed deep learning screening system’s efficacy against other state-of-the-art approaches like U-Net, SegNet, and a previous method. The creators shared that their suggested technique obtained a precision of 0.93, recall of 0.90, F-measure of 0.88, and Jaccard index of 0.84 on a 143-WSI dataset, demonstrating remarkable performance over the other methods. Additionally, the proposed method demonstrated a remarkable processing speed of 210 s per WSI, which is 20 times and 19 times faster than U-Net and SegNet, respectively. The potential for an AI system to produce these exceptional results and suggest a method for quickly and accurately identifying severe cervical pathologies in real-world clinical environments could be highly advantageous for the medical community, particularly in areas with limited resources. The results of this analysis demand additional inquiry and authentication in larger and more varied datasets [20].

Kanavati and team designed a model for detecting cervical cancer in liquid cytology WSIs that utilized deep learning, consisting of trained convolutional and recurrent neural networks. The model was tested on 1605 training and 1468 multi-test set WSIs and achieved an ROC AUC range of 0.89–0.96, indicating its potential to assist in screening. Furthermore, the model generates neoplastic cell probability heatmaps that help identify suspicious regions. The model exhibited either comparable or superior accuracy, sensitivity, and specificity when compared with semi-automated devices. Therefore, it has the potential to standardize screening and reduce fatigue. The results of this study suggest that incorporating deep learning into cervical cancer screening could have a substantial impact on the accuracy and efficiency of the screening process. Furthermore, the integration of this model into healthcare may lead to the premature detection of cervical cancer and potentially rescue lives [21].

Hamdi and colleagues developed a novel approach for the analysis of whole slide cervical images and cancer staging using a hybrid deep learning system to generate a combination of models that includes ResNet50, VGG19, GoogLeNet, Random Forest, and support vector machine. The team also utilized the Active Contour Algorithm for segmentation and fused deep model features as another approach. The ResNet50-VGG19-Random Forest model achieved outstanding results on a dataset of 962 cervical squamous cell images. Particularly, the prototype accomplished 97.4% sensitiveness, 99% exactness, 99.6% exactitude, 99.2% selectivity, and 98.75% AUC, which displays noteworthy potential for beforehand detection. Considering the encouraging outcomes of this research, it is probable that upcoming studies may comprise clinical data and more comprehensive, diverse datasets, as well as an investigation of further deep learning models. Overall, this proposed plan of action has the potential to advance the field of cervical cancer diagnosis and improve patient outcomes [22].

Diniz and colleagues conducted a study that involved comparing ten deep convolutional neural networks for classifying cervical cells in PAP smears into two, three, and six categories using conventional cytology images. They proposed an ensemble of the top three models and used extensive data augmentation and balancing. The appraisal of the investigation utilized accuracy, specificity, F1-score, cross-validation, recall, and precision. The ensemble model outperformed individual and prior architectures across all classification tasks. The recall achieved for the two, three, and six classes were 0.96, 0.94, and 0.85. The study highlights the potential of ensemble deep learning in improving the accuracy of cervical cancer screening and, subsequently, patient outcomes. The findings of the investigation suggest that the utilization of an assembly model may result in improved efficacy when compared with individual models. The authors further established the efficacy of enhancing data through augmentation and balancing to enhance the precision of the model. This study provides valuable insights into the use of advanced ensemble deep learning in cervical cancer screening and has the potential to inspire further research in this field [23].

Tripathi and colleagues conducted a study that involved the classification of five cervical cancer cell types in 966 PAP smear images using four pre-trained deep models. These architectures were constructed by a team of highly skilled engineers and were carefully tested to ensure optimal performance. ResNet-152 reached the maximum precision of 94.89%, with VGG-19, ResNet-50, and VGG-16 close on its heels. Furthermore, the study reported class-wise performance, and certain combinations of models achieved 100% recall and precision for specific classes. The investigation accentuates the potential of deep transfer learning for precise classification and implies that further progressions in original models, hyperparameter optimization, and clinical data integration could boost the accuracy even further. In line with the data, this analysis provides valuable insights for future research in the field of cervical cancer grouping. These findings can contribute to improving cervical cancer diagnosis and patient outcomes [24].

Zhou and colleagues presented a comprehensive cervical screening framework that includes three stages: cell detection, image classification, and case classification. The first stage involved detecting cells using the RetinaNet model, which achieved an impressive 0.715 average precision in just 0.128 s per image. The following step integrated an innovative patch encoder-fusion component for image classification, achieving a 0.921 accuracy and 0.903 sensitivity. For the final phase, a support vector machine functioned as a case classifier, providing an accuracy of 0.905 and sensitivity of 0.891, outclassing other models. These numerical results clearly demonstrate the framework’s effectiveness in leveraging cell cues to improve the robustness of case diagnosis. In general, this inventive system shows vast potential for enhancing the detection and diagnosis of cervical cancer, ultimately resulting in improved health outcomes for women. Additional exploration is warranted to affirm the framework’s effectiveness in larger and more diverse patient populations [25].

The CervixFormer proposal has demonstrated a considerable level of efficacy in the classification of PAP smear whole slide images. Unlike other inferior transformer and convolutional models, this particular model has exhibited commendable performance on both private six-class and public four-class datasets. Additionally, the program has showcased strong binary, three-class, and five-class cellular classification precision, recall, accuracy, and F1-scores. The CervixFormer proposal has utilized data augmentation and stain normalization techniques to enhance diversity and staining invariance across the datasets. The incorporation of Swin Transformer subnetworks into the model has facilitated multi-scale feature learning through the fuzzy rank fusion approach. Consequently, the GradCAM visualizations on the important regions have provided clinical interpretability of the model’s outputs. Overall, the CervixFormer proposal has shown promise as a scalable and reliable solution for cervical screening and diagnosis, with the potential for clinical deployment [26].

In brief, the available data suggest that AI exhibits remarkable detection rates and precision when it comes to cytology. Nonetheless, there remains the potential for conducting extensive research and delving into innovative applications within this field. For instance, the development of AI microscopes can potentially revolutionize cytology screening by enhancing its efficiency and accuracy. Additionally, an AI assistant colposcopy represents a highly advanced instrument that can assist in the identification and evaluation of cervical cancer [27]. This particular innovation possesses the capacity to transform the realm of cervical cancer treatment, as it has the ability to furnish healthcare practitioners with invaluable perspectives and assistance. Various potential paths regarding the treatment of cervical cancer exist, highlighting immunotherapy, targeted therapy, and the use of PARP inhibitors [28].

**Table 1:** Summary of remarkable works focusing on AI-assisted PAP smear (* Please see footnote for abbreviation and Appendix A for details).

References	Year	Datasets (Number of Images)	Metrics	Methods
Chankong et al. [8]	2014	ERUDIT (552) Herlev (917)	Accuracy 93.78 to 99.27%	Bayesian classifier * + KNN * + ANN *
Wang et al. [11]	2019	Private (362)	Sensitivity 94.25%; specificity 93.45%	Mean-Shift clustering algorithm *
Zhang et al. [13]	2017	Herlev (917) HEMLBC (2370)	Accuracy 98.30 to 98.6%; specificity 98.30 to 99.00%	CNN * + transfer learning *
Shi J et al. [14]	2021	SIPAKMeD (4049)	Accuracy 98.37%; sensitivity 99.80%	CGN *
Bao et al. [15]	2020	Cervical cancer screening program (703,103)	CIN1+ Sensitivity 88.9%; specificity 95.8%; CIN2+ Sensitivity 90.10%; specificity 94.80%	DL *
Zhu et al. [16]	2021	Cytological image biopsy diagnosis proven (980)	Sensitivity 94.74%	AIATBS *
Chen et al. [17]	2021	WSI (198,952)	Accuracy 88.30%; sensitivity 92.83%; specificity 91.03%; precision 82.26%; F1-score 87.04%	CompactVGG *
Wei et al. [18]	2021	WSI (2019)	Accuracy 80.80%; sensitivity 90.60%; specificity 71.00%	YOLCO *
Cheng et al. [19]	2021	WSI (3545)	Sensitivity 93.50%; specificity 95.10%	RNN *
Wang et al. [20]	2021	WSI (143)	Precision 93.00%; recall 90.00%; F1-score 88.00%	FCN *
Kanavati et al. [21]	2022	WSI (1605)	Accuracy 90.00%; sensitivity 86.00%; specificity 91.00%	CNN + RNN
Hamdi et al. [22]	2023	WSI (962)	Accuracy 99.00%; sensitivity 97.40%; specificity 99.20%; precision 99.60%	RF * + ResNet50 * + VGG19 *
Diniz et al. [23]	2021	CRIC (3233)	Accuracy 96.00%; recall 94.00%; specificity 97.00%; precision 94.00%; F1-score 94.00%	MobileNet * + InceptionNet * + EfficientNet *
Tripathi et al. [24]	2021	SIPAKMED (966)	Accuracy 94.89%	ResNet-152 *
Zhou et al. [25]	2021	WSI (237)	Accuracy 90.50%; sensitivity 89.10%; F1-score 86.70%	SVM * + RetinaNet * + Encoder *
	2023	Mendeley (963) SIPaKMeD (4049) Dankook University Hospital (100,000) AI-Hub (20,000)	Accuracy 95.00%; recall 95.00%; precision 97.00%; F1-score 95.00%	GRAD-CAM * + Swin Transformer *
Khan et al. [26]				

* KNN: K-nearest neighbor; ANN: artificial neural network; CNN: convolutional neural network; CGN: convolutional graph network; DL: deep learning; AIATBS: Artificial Intelligence-Assisted ThinPrep^®^ Imaging System; YOLCO: You Only Look Cytopathology Once; RNN: recurrent neural network; FCN: fully convolutional network; RF: Random Forest; SVM: support vector machine; GRAD-CAM: Gradient-weighted Class Activation Mapping.

## 3. Discussion

The detection and treatment of cervical cancer, a key area of women’s healthcare, can be transformed by the implementation of AI. The current manual screening methods, such as the PAP smear test, are limited by the complexity, monotony, and subjectivity involved in the human examination of cytology slides, leading to inefficiency and inaccuracy. To overcome these limitations, computer-aided diagnosis leverages advanced algorithms to analyze cell morphology and classify smears quickly. AI can enhance the accuracy and specificity of screening and diagnostic programs, overcome time constraints, and prevent bias caused by subjective factors. By allowing cervical cancer screening to be executed in areas with limited resources, artificial intelligence has the potential to significantly decrease the prevalence of cervical cancer.

The employment of human intelligence suggests multiple challenges that require attention to adequately integrate artificial intelligence (AI) algorithms. A major obstacle is the insufficiency of information, which often requires millions of data points for AI to achieve satisfactory efficacy levels. Unfortunately, current clinical data may lack markers, have uncertain quality, and be scarce, making it difficult to manage medical data effectively. It is critical to establish standardized and extensive databases in the future while considering data security concerns and over-fitting, which can lead to over-diagnosis. Clinical practice consistently demonstrates the reliability of AI-based models. However, it is noteworthy that AI is not designed to supplant clinicians but rather to function as a supportive diagnostic tool. Furthermore, AI may result in system paralysis, necessitating technical skills for maintenance and specialized training, and systems must be implemented to ensure proper maintenance.

Recent developments in the field of deep learning have generated a renewed sense of optimism with regards to achieving dependable automated screening. Unlike human observers, AI algorithms have the ability to analyze large datasets and identify subtle patterns or anomalies, resulting in more accurate diagnoses or outcomes. Additionally, AI has the potential to improve patient comfort and well-being by utilizing less invasive procedures for detecting cancer cells from medical images. These advantages highlight the significant potential of AI in the field of medical imaging and diagnosis, and underscore the importance of continued research and development in this area. The amalgamation of AI into cervical screening offers an array of advantages like increased diagnostic precision, efficiency, enhanced patient comfort, decreased healthcare expenses, and superior outcomes. According to a cost-effectiveness analysis by Chen et al., AI-based PAP smear screening can save up to 30% of healthcare costs compared with conventional cytology screening [17].

Nevertheless, the identification of these prospects is met with impediments such as ensuring the precision of data, obtaining regulatory approval, addressing ethical considerations, clinically validating the functionality of systems, and encouraging patient acceptance. Moreover, obstructions persist, encompassing inadequate labeled data, variations in staining, the opacity of the model, and the requisite compliance with regulatory and ethical standards. To corroborate the efficacy of artificial intelligence as a screening adjunct, further extensive and comprehensive exploration in genuine, real-life scenarios is imperative.

The field of AI-assisted PAP smear classification research has a lengthy history in comparison to other domains, and its progress has nearly plateaued due to the accumulation of findings. Nonetheless, AI-based methods have not gained widespread use in clinical practice, and we contend that three obstacles must be surmounted. Firstly, the accurate classification of PAP smears necessitates the analysis of WSIs, which, given that diagnostic patches compose only a small proportion of tens of thousands of image patches in a slide, demands high-throughput and precise analysis of thousands of patches from WSIs. Secondly, the scanning of digital slides, which necessitates high-performance and high-cost devices, is required for WSI analysis, and their global penetration remains limited due to pricing. Finally, the absence of medical insurance support to subsidize the costs of expensive digital scanners required to address the second challenge and the high-end GPU necessary to tackle the first challenge present daunting obstacles to clinical adoption. In short, AI-based PAP smear screening is the processing of large-scale and high-resolution WSIs, which require high-performance and high-cost devices and GPUs. To overcome this challenge, we used a novel image analysis method based on object segmentation and CNN that can efficiently segment and quantify cells in WSIs without compromising accuracy.

In brief, the technical performance of AI-based techniques is commendable; however, there are still economic and infrastructure challenges that need to be addressed for these techniques to have a widespread impact in domains such as PAP smear screening. Artificial intelligence may have applications beyond early screening and diagnosis, including the prediction of prognosis, prevention, and treatment of cervical cancer. Additional inquiry is required to be performed on the subject of therapy and prognosis for the purpose of efficient therapy selection and to enable the global elimination of cervical cancer. As the incidence of cervical adenocarcinoma and other rare pathological types increases, AI should be utilized for early diagnosis in the future. Furthermore, AI can be utilized for the noninvasive differentiation of cervical cancer from other diseases. With the additional advancement of AI technologies, the estimation of cervical cancer can be significantly improved, leading to noteworthy enhancements in cervical cancer screening and diagnosis, refinement of staging systems, and better patient prognosis.

## 4. Conclusions

The execution of AI in the screening of cervical cancer provides a hopeful alteration in the detection and management of this ailment. The current screening procedures, such as PAP smears, possess insufficiencies with regard to their effectiveness and precision. PAP smears require manual examination, which can be intricate, tedious, and subject to human subjectivity and fatigue. Nevertheless, the utilization of computer-aided diagnosis strives to surmount these limitations by implementing sophisticated algorithms that can swiftly evaluate cell morphology and classify slides on the basis of the probability of abnormality or malignancy. With recent advancements in deep learning, there is newfound hope in achieving the long-awaited goal of dependable automated screening. The utilization of AI algorithms is capable of analyzing tremendous datasets and detecting patterns and anomalies that may go unnoticed by human observers. Undoubtedly, this technological innovation has the potential to significantly improve patient outcomes by enabling more precise diagnoses and treatments. As AI and other advanced machine learning techniques continue to evolve and improve, they offer promising new avenues for enhancing the accuracy and efficiency of medical imaging and diagnosis, ultimately leading to better patient care and outcomes.

Additionally, the utilization of AI screening methods holds the possibility of mitigating the need for invasive procedures, thereby reducing patient discomfort and enhancing patient results. Also, AI-driven PAP smear diagnostics may serve as a telemedicine and mobile health system, simplifying remote and accessible screening and diagnosis of cervical cancer. Moreover, AI-based PAP smear diagnostics can function as a research and education system that can foster the dissemination and advancement of knowledge and skills in cervical cancer. The implementation of AI for the purpose of identifying cervical cancer is hindered by various obstacles and challenges, such as deficient data quality, lack of regulatory authorization, ethical concerns, clinical validation, and patient skepticism despite its vast potential for significant gains. The potential of AI in revolutionizing cervical cancer screening programs worldwide through the efficient use of digitized cytology is considerable, but it will require careful consideration and effective action to overcome the challenges and limitations.

Effective integration of workflow and ongoing model refinement is essential to fully capitalize on the benefits of AI while minimizing risks. Although there are still challenges, evidence indicates that AI could revolutionize the detection of cervical cancer and other illnesses in the upcoming years. However, AI-assisted cytologic evaluation should not replace human governance and accountability. Rather, it should function as a tool to enhance human abilities and enhance patient outcomes.

## Figures and Tables

**Figure A1:**
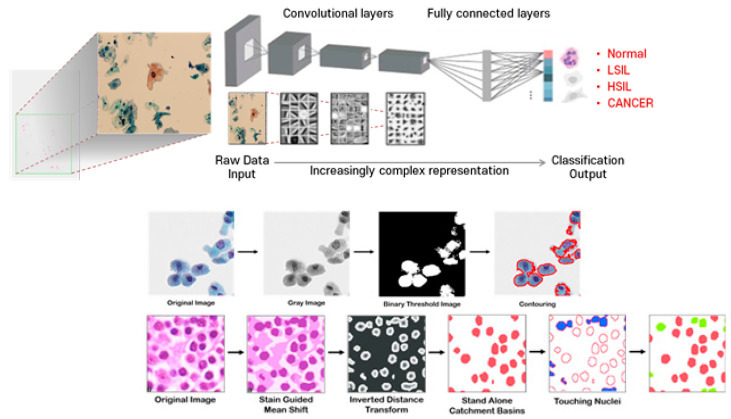
Object Segmentation for Accurate Cell Segmentation and Quantification in Cytology using CNN: Schematic representation of CNN and object segmentation for cell segmentation and quantification. CNNs use convolution instead of general matrix multiplication in at least one of their layers. A typical CNN architecture consists of three layers: a convolutional layer, a pooling layer, and a fully connected layer. The convolutional layer applies filters to the input image to extract features, the pooling layer downsamples the image to reduce computation, and the fully connected layer makes the final prediction. Object segmentation, a computer vision technique, is used to accomplish cell segmentation and quantification. This process involves dividing the cells within patches, which allows physicians to analyze and scan each cell if necessary. By utilizing object segmentation, physicians can more accurately identify and quantify cell types and their distribution, leading to more accurate diagnoses in cytology (illustrated by authors).

## Data Availability

Some or all datasets generated during and/or analyzed during the current study are not publicly available but are available from the corresponding author upon reasonable request.

## Appendix A

1. Bayesian classifier* is a classification algorithm that uses Bayes’ theorem to make predictions based on the probability of an event occurring given certain evidence. It calculates the probability of a particular class or category based on the presence of certain features or attributes. The algorithm assumes that the features are independent of each other, and it updates the probability estimates as new evidence is observed. Bayesian classifiers have been applied in the field of medical diagnostics, including cervical cancer screening and diagnosis. They have been used in integrated classifiers designed for cervical cell classification, achieving high accuracy at both the smear and cell levels. Bayesian classifiers are one of the techniques used in the automatic analysis of cervical smears, particularly in the segmentation and classification stages, to improve screening efficiency.
2. KNN (K-nearest neighbor) is a classification algorithm that is commonly used in machine learning. It is a non-parametric algorithm that makes predictions based on the similarity of a new data point to its k nearest neighbors in the training dataset. The algorithm calculates the distance between the new data point and the existing data points and assigns the new data point to the class that is most common among its k nearest neighbors. KNN has been used in the field of medical image analysis, including in the automatic analysis of cervical smears for cervical cancer screening. It has been applied in the segmentation and classification stages of the analysis process to improve screening efficiency. KNN is one of the techniques used in the integrated classifiers designed for cervical cell classification, contributing to high accuracy in both smear and cell-level classification.
3. An artificial neural network (ANN) is a type of machine learning process that uses interconnected nodes or neurons in a layered structure that resembles the human brain. ANNs are computing systems inspired by the biological neural networks that constitute animal brains. They are composed of artificial neurons which are conceptually derived from biological neurons. Each artificial neuron has inputs and produces a single output which can be sent to multiple other neurons. The inputs can be the feature values of a sample of data, and the output can be a prediction or classification. Artificial neurons are software modules, called nodes, and artificial neural networks are software programs or algorithms that, at their core, use computing systems to solve mathematical calculations. ANNs are a subset of machine learning and are at the heart of deep learning. They create an adaptive system that computers use to learn from their mistakes and improve continuously.
4. The mean-shift clustering algorithm is a non-parametric, density-based clustering algorithm used in unsupervised learning to identify clusters in a dataset. It is a mode-seeking algorithm that assigns data points to the clusters iteratively by shifting points towards the highest density of data points in the region. Unlike K-means clustering, it does not make any assumptions and does not require the number of clusters to be specified in advance. The algorithm iteratively performs these shifts until the points converge to a local maximum of the density function. The mean-shift clustering algorithm is widely used in real-world data analysis, such as image segmentation, because it is non-parametric and does not require any predefined shape of the clusters in the feature space.
5. A convolutional neural network (CNN) is a type of deep learning neural network architecture that is commonly used in computer vision. It is specifically designed to process pixel data and is used in image recognition and processing. CNNs use a mathematical operation called convolution in place of general matrix multiplication in at least one of their layers. The architecture of a CNN typically has three layers: a convolutional layer, a pooling layer, and a fully connected layer. The convolutional layer applies filters to the input image to extract features, the pooling layer downsamples the image to reduce computation, and the fully connected layer makes the final prediction. CNNs are often used in image recognition systems and have applications in various fields such as self-driving cars, facial recognition, and natural language processing* (Figure A1).

6. Transfer learning is a machine learning technique that involves using a pre-trained model on one task and applying it to a related task to achieve better performance. It is a way to leverage the knowledge and features learned from one problem to solve another related problem. Transfer learning is commonly used in deep learning, especially in computer vision and natural language processing tasks. By using transfer learning, one can save time and computational resources by reusing the pre-trained model’s weights and parameters instead of starting from scratch. Transfer learning can be used to improve the performance of a model when there is a limited amount of labeled data available for the new task. However, transfer learning may not work if the high-level features learned by the pre-trained model are not sufficient to differentiate the classes in the new problem.
7. CGN stands for convolutional graph network. It is a type of artificial neural network that is used in the field of cervical cancer screening and diagnosis. The CGN model is a part of the workflow in colposcopy image classification, which aims to improve the consistency between colposcopy and pathology results. Colposcopy, performed by trained clinicians, is an important step in cervical cancer screening. However, the consistency between colposcopy and pathology can be poor, leading to potential misdiagnosis and missed diagnosis. The CGN model, along with other techniques such as convolutional neural networks (CNNs) and hybrid deep feature fusion (HDFF) techniques, is used to classify colposcopy images accurately and improve the diagnostic accuracy in cervical cancer screening.
8. DL stands for deep learning. It is a technology that has been widely used in medical imaging, including in the field of cervical cancer screening and diagnosis. DL models, such as convolutional neural networks (CNNs), have been developed to improve the accuracy of cervical cancer diagnosis. These models use advanced algorithms to analyze and classify colposcopy images, helping to overcome the limitations of traditional colposcopy methods. DL-based classifiers have shown promising results in accurately classifying cervical cells and guiding biopsy procedures. They have also been used to grade colposcopy impressions and improve the consistency between colposcopy and pathological results. DL technology has the potential to enhance the diagnostic performance of cervical cancer screening and improve patient outcomes.
9. AIATBS stands for Artificial Intelligence-Assisted ThinPrep^®^ Imaging System. It is a technology that has been widely used for HPV testing and cytology in cervical cancer screening. The AIATBS has shown good detection rates and accuracy in detecting cervical intraepithelial neoplasia (CIN) and other cervical lesions. It has been used in clinical prospective validation studies and has demonstrated high sensitivity in detecting CIN. Additionally, AI microscopes with augmented reality (AR) display have been developed, which significantly improve detection sensitivity for low squamous intraepithelial lesion (LSIL) and high-grade squamous intraepithelial lesion (HSIL) recognition. The AIATBS technology has the potential to enhance the accuracy and consistency of cervical cytology screening, and it is being further studied and applied in various research studies and applications.
10. CompactVGG is a deep learning model used in cervical cancer screening. It is a modified version of the VGG16 model, designed to reduce calculation costs while maintaining classification performance. CompactVGG has a narrower and shallower architecture compared with VGG16, with fewer convolution filter channels and fewer convolution and fully-connected layers. This reduction in complexity helps to improve the efficiency of the model in terms of both speed and computational resources. CompactVGG has been shown to achieve high scores and superior performance in the classification of cervical cells, including screening positive cervical cells, negative cervical cells, and junk cells. The model incorporates regularization techniques such as dropout and L2-norm regularization, as well as data augmentation approaches to enhance classification performance.
11. YOLCO (You Only Look Cytopathology Once) is a framework proposed based on the YOLOv3 model for representing whole slide images (WSIs) in cytopathology. It utilizes feature vectors corresponding to small areas within the WSI to improve effectiveness and efficiency. YOLCO employs a fully convolutional structure with a lightweight design to avoid repeated computations and increase input size. It incorporates a multi-task supervision approach, combining classification and location tasks, to establish feature mixtures with both semantic and location information. YOLCO demonstrates robustness in encoding WSIs and outperforms other models in different settings of WSI datasets. However, it may not perform as well with natural data. The proposed framework addresses challenges in WSI representation and enhances the generalization of the model for cytopathology applications.
12. A recurrent neural network (RNN) is a type of neural network that is designed to process sequential data or time series data. Unlike traditional neural networks, RNNs have a “memory” that remembers information about a sequence by using a hidden layer. The output from the previous step is fed as input to the current step, allowing the network to remember previous inputs and produce outputs that depend on the previous inputs. RNNs are used in various applications such as speech recognition, natural language processing, and handwriting recognition. They are also theoretically Turing complete, which means they can run arbitrary programs to process arbitrary sequences of inputs. RNNs can handle input sequences of variable length, making them well suited for tasks such as speech recognition and natural language processing.
13. FCN stands for fully convolutional network. It is a type of neural network architecture commonly used in computer vision tasks, including image segmentation. FCN replaces the fully connected layers of traditional convolutional neural networks (CNNs) with convolutional layers to enable end-to-end pixel-wise predictions. This allows FCN to take an input image of any size and produce a corresponding output map with pixel-level predictions. FCN has been applied in various medical imaging tasks, including cervical cancer screening and diagnosis. In the context of cervical cancer screening, FCN has been used for tasks such as cell segmentation and classification, improving the accuracy and efficiency of the screening process. FCN is particularly useful in handling large-scale whole slide images (WSIs) in cytopathology, where it can encode WSIs effectively and enhance the generalization of the model.
14. Random Forest is a machine learning algorithm that combines the predictions of multiple decision trees to make more accurate predictions. It is an ensemble learning method that uses a collection of decision trees, where each tree is trained on a different subset of the data and features. The final prediction is made by aggregating the predictions of all the individual trees. Random Forest is known for its ability to handle high-dimensional data, handle missing values, and reduce overfitting. It is widely used in various domains, including healthcare and medical research. In the context of cervical cancer screening and diagnosis, Random Forest can be applied to analyze and classify cervical cells in smears, improving the accuracy and efficiency of the screening process.
15. ResNet50 is a deep convolutional neural network architecture that was introduced by Microsoft Research in 2015. It is a variant of the ResNet (Residual Network) model, which is known for its ability to train very deep neural networks effectively. ResNet50 specifically refers to a ResNet model with 50 layers. It is widely used in computer vision tasks, including image classification and object detection. ResNet50 incorporates skip connections, or shortcuts, that allow the network to learn residual mappings, making it easier to train deeper networks without suffering from the vanishing gradient problem. This architecture has been applied in various medical imaging tasks, including the analysis of whole slide images in cervical cancer screening. ResNet50 has demonstrated excellent performance in image recognition tasks and has become a popular choice in the deep learning community.
16. VGG19 is a deep convolutional neural network architecture that was introduced by the Visual Geometry Group (VGG) at the University of Oxford. It is a variant of the VGG model, which is known for its simplicity and effectiveness in image classification tasks. VGG19 specifically refers to a VGG model with 19 layers, including convolutional layers, pooling layers, and fully connected layers. It has a uniform architecture with small 3 × 3 filters and max pooling layers. VGG19 has been widely used as a benchmark model in computer vision research and has achieved excellent performance in image recognition tasks. It has also been applied in medical imaging tasks, including the analysis of cervical cell images in cervical cancer screening. VGG19’s deep architecture allows it to learn complex features from images, making it a powerful tool for image classification tasks.
17. MobileNet is a type of convolutional neural network architecture designed for mobile and embedded vision applications. It is based on a streamlined architecture that uses depthwise separable convolutions to build lightweight deep neural networks that can have low latency for mobile and embedded devices. MobileNet is Tensorflow’s first mobile computer vision model. MobileNetV1 is the original version of MobileNet, while MobileNetV2 is similar to the original but uses inverted residual blocks with bottlenecking features and has a lower parameter count. MobileNetV3 is the latest version of MobileNet and has improved accuracy and speed compared with the previous versions. MobileNets are small, low-latency, low-power models that can be used for classification, detection, and other common tasks in which convolutional neural networks are effective.
18. InceptionNet is a convolutional neural network architecture developed by Google to improve the performance of previous convolutional neural networks on the ImageNet Large Scale Visual Recognition Challenge (ILSVRC) benchmark. It is known for using “inception modules,” which are blocks of layers designed to learn a combination of local and global features from the input data. InceptionNet won the 2014 ILSVRC competition and has been used in various applications, including image classification, object detection, and image segmentation. InceptionNet is also referred to as GoogLeNet or Inception v1, and it has been updated to Inception v2, v3, and v4, with each version improving on the previous one. The design of InceptionNet was intended to allow deeper networks while also keeping the number of parameters from growing too large. The architecture uses a combination of 1 × 1, 3 × 3, and 5 × 5 convolutions on the input data and utilizes auxiliary classifiers to improve performance. InceptionNet is a deep classifier that has been subject to the vanishing gradient problem, which can be addressed by using batch normalization.
19. EfficientNet is a convolutional neural network architecture and scaling method that uniformly scales all dimensions of depth, width, and resolution using a compound coefficient. It is designed to improve the efficiency of existing ConvNets based on the available resources such as memory and FLOPS. The authors of EfficientNet applied the compound scaling method to the baseline network EfficientNet-B0 by setting Φ = 1 and doing a grid search to find the parameters α, β, and γ based on the equations given in the previous section and under the constraint α.β².γ² ≈ 2. The results were α = 1.2, β = 1.1, and γ = 1.15, and the network’s dimension equation was used to obtain a family of neural networks, EfficientNet-B1 to B7. EfficientNet has achieved state-of-the-art accuracy with up to 10x better efficiency (smaller and faster) and has been compared with other existing CNNs on ImageNet. The EfficientNet models achieve both higher accuracy and better efficiency over existing CNNs, reducing parameter size and FLOPS by an order of magnitude. EfficientNet is taken even further with EfficientNetV2, which is even more powerful.
20. ResNet-152 is a variant of the ResNet architecture that has 152 layers. It was introduced in the original ResNet paper by He et al. in 2015. ResNet-152 is a deep residual neural network that uses skip connections to address the vanishing gradient problem that occurs in deep neural networks. The skip connections allow the network to learn residual functions instead of directly learning the underlying mapping, which makes it easier to train deeper networks. ResNet-152 has achieved state-of-the-art performance on various computer vision tasks, including image classification, object detection, and semantic segmentation. However, ResNet-152 has a large number of parameters, which makes it computationally expensive to train and deploy.
21. Support vector machine is a machine learning algorithm used for classification and regression tasks. It works by finding an optimal hyperplane that separates different classes in the data. SVM aims to maximize the margin between the hyperplane and the nearest data points, which helps in achieving better generalization and reducing overfitting. It can handle both linear and non-linear data by using different kernel functions. SVM has been widely used in various applications, including image classification, text classification, and bioinformatics. It has shown good performance in tasks such as cancer diagnosis and gene expression analysis. SVM is known for its ability to handle high-dimensional data and its robustness against noise.
22. RetinaNet is a one-stage object detection model that utilizes a focal loss function to address class imbalance during training. It is a single, unified network composed of a backbone network and two task-specific subnetworks. RetinaNet uses a feature pyramid network to efficiently detect objects at multiple scales and introduces a new loss, the focal loss function, to alleviate the problem of the extreme foreground–background class imbalance. RetinaNet has become a popular object detection model to be used with aerial and satellite imagery. It was formed by making two improvements over existing single-stage object detection models—Feature Pyramid Networks (FPNs) and Focal Loss. The RetinaNet model has separate heads for bounding box regression and for predicting class probabilities for the objects.
23. Encoder is a term commonly used in the field of machine learning and artificial intelligence. It refers to a component or algorithm that is responsible for transforming input data into a different representation or feature space. In the context of the provided sources, there is no specific mention of an encoder. However, there are references to different stages and techniques used in the analysis of cervical cancer screening, such as image acquisition, preprocessing, segmentation, feature extraction, and classification. These stages involve various algorithms and methods that may include encoding or transforming the input data to extract relevant features for classification or analysis purposes. While the term “encoder” is not explicitly mentioned, the overall process of analyzing cervical cancer screening data involves multiple steps that may include encoding or transforming the data in some way.
24. GRAD-CAM (Gradient-weighted Class Activation Mapping) is a technique used in computer vision and deep learning to visualize the regions of an image that are important for a neural network’s prediction. It helps to understand which parts of the image contribute the most to the network’s decision-making process. GRAD-CAM generates a heatmap that highlights the regions of the image that are most relevant to the predicted class. It achieves this by computing the gradients of the target class with respect to the feature maps of the last convolutional layer in the network. The gradients are then used to weight the feature maps, resulting in a heatmap that indicates the importance of each pixel in the image. GRAD-CAM has been applied in various medical imaging tasks, including cervical cancer screening, to provide insights into the decision-making process of deep learning models.
25. The Swin Transformer is a deep learning model architecture that uses shifted windows to limit the computation required for self-attention. It is a transformer-based model that builds hierarchical feature maps by merging image patches in deeper layers. The Swin Transformer is highly efficient and has greater accuracy than the Vision Transformer (ViT). The Swin Transformer is used as the backbone in many vision-based model architectures today. The Swin Transformer introduced two key concepts to address the issues faced by the original ViT—hierarchical feature maps and shifted window attention. The Swin Transformer is a type of one-stage object detection model that utilizes a focal loss function to address class imbalance during training. It is a single, unified network composed of a backbone network and two task-specific subnetworks. The Swin Transformer has been used in various applications, including image classification, object detection, and semantic segmentation.
